# Determinants of smoking cessation program completion and continued tobacco use in a referral program in West Virginia: An EHR data analysis from 2018–2023

**DOI:** 10.18332/tpc/221805

**Published:** 2026-07-09

**Authors:** Maryam Pathan, Marquis Demniak, Sabina O. Nduaguba

**Affiliations:** 1 Department of Pharmaceutical Systems and Policy, School of PharmacyWest Virginia UniversityMorgantownUnited States; 2 West Virginia University Cancer InstituteMorgantownUnited States

**Keywords:** smoking cessation, tobacco use, program completion, electronic health records, West Virginia

## Abstract

**Introduction:**

Smoking is a risk factor for various comorbidities, yet only a few who attempt to quit are successful. This study examined factors associated with program completion (defined as at least four clinic visits) of a tobacco cessation program in West Virginia and identified predictors of continued tobacco use at the last visit.

**Methods:**

An observational retrospective cohort study analyzed electronic health records (2018–2023) collected from a tobacco cessation clinic. Patients aged ≥18 years with a history of tobacco use received individualized treatment combining pharmacological and behavioral therapy. Follow-up visits assessed cessation progress. Logistic regression estimated odds ratios (ORs) and 95% confidence intervals for predictors of program completion and continued tobacco use.

**Results:**

Among 407 participants, 85% completed at least four clinic visits, but 81.57% continued tobacco use at the last visit. Cancer (AOR=2.77; 95% CI: 1.36–5.65) and hypertension (AOR=3.26; 95% CI: 1.64–6.47) were associated with higher odds of program completion, whereas alcohol use was associated with lower odds of program completion (AOR=0.52; 95% CI: 0.29–0.93). Continued tobacco use was associated with higher tobacco pack-years (AOR=1.01; 95% CI: 1.0–1.02) and lower number of clinic visits (AOR=0.98; 95% CI: 0.96–0.99).

**Conclusions:**

Alcohol use, cancer, and hypertension were predictors of program completion, while continued tobacco use on the last visit was associated with tobacco pack-years and the number of clinic visits. Despite high program completion, most participants continued using tobacco on the last visit, highlighting the persistent challenge with the poor effectiveness of smoking cessation treatments. Integrating tailored cessation strategies and utilizing chronic diseases as motivational tools could improve the effectiveness of interventions in high-prevalence states like West Virginia.

## Introduction

Cigarette smoking is responsible for more than 480000 preventable deaths per year in the US^1^. West Virginia is one of the 12 states within the Midwest and South region, collectively known as the ‘Tobacco Nation’, that have a 40% higher average smoking rate than the other states in the US^2^ and has the second-highest prevalence of cigarette smoking nationwide. Twenty-three percent of adults in West Virginia are cigarette smokers, which is higher than the national rate of 15.5% in 2020^2,3^. Due to the high prevalence of smoking in West Virginia, the state also reports a higher prevalence of smoking-related diseases, such as arthritis (38.9%), COPD (13.9%), cardiovascular disease (14.6%), diabetes (15%), and cancer (14%)^3^. About 44% of the people who smoke tobacco try to quit each year, but only 3–6% achieve cessation^4^. According to the Behavioral Risk Factor Surveillance System report, an estimated 47% of the current adult smokers in West Virginia reported quitting smoking for at least 1 day in 2019^4^. While access to quitting services helps reduce tobacco use and encourage quitting, it is challenging to promote and ensure adherence to these services among people who smoke tobacco. A US-based study reported that fewer than four in ten adults who made a quit attempt or quit smoking used evidence-based treatment (counseling and/or medication) to help with smoking cessation^5^.

Numerous studies have assessed factors associated with successful tobacco cessation in the US and other countries^6-10^. For example, Lee et al^6^. reported that successful quitters were more likely to have rules against smoking in their homes, to be aged ≥35 years, to be married or living with a partner, to be non-Hispanic White, and to have at least a college education than those who could not quit smoking. Studies have also reported the predictors of smoking cessation in clinic-based programs. A US study from 2006 reported that older age, having more than two children, low socioeconomic status, and severity of nicotine dependence were predictors of successful cessation in a clinic^11^. Other studies have also identified specific comorbidities associated with smoking prevalence and cessation failures, highlighting the role of chronic conditions in smoking-related behavior^12,13^.

While several studies reported on factors associated with tobacco cessation, few have examined predictors of retention and completion of clinic-based cessation programs in the US^14^. Although literature on cessation in non-clinic settings, such as state quitlines and digital health programs, exists, these findings might not be applicable to the clinic-based cessation programs^15,16^. Existing studies of cessation programs in clinic settings are limited, with relatively high retention rates of about 80% reported^17^. Building on this limited but growing body of evidence, the present study aims to address two questions in a West Virginia state-based smoking cessation clinic. Using a retrospective cohort design, the study aims to determine: 1) the predictors of completion of the tobacco cessation program, defined as completing at least four visits to the cessation clinic; and 2) the predictors of smoking cessation failures, defined as continued tobacco use at the last visit to the cessation program.

## Methods

### Data source and setting

This study was based on electronic health records (EHRs) of patients referred to the tobacco cessation clinic within a single academic medical center in West Virginia between 2018 and 2023. The West Virginia Clinical and Translational Science Institute (WVCTSI) provided the data, and the study was approved by the West Virginia University Institutional Review Board (Protocol #: 2206588779; 17 June 2022).

### Study design

**Figure 1:**
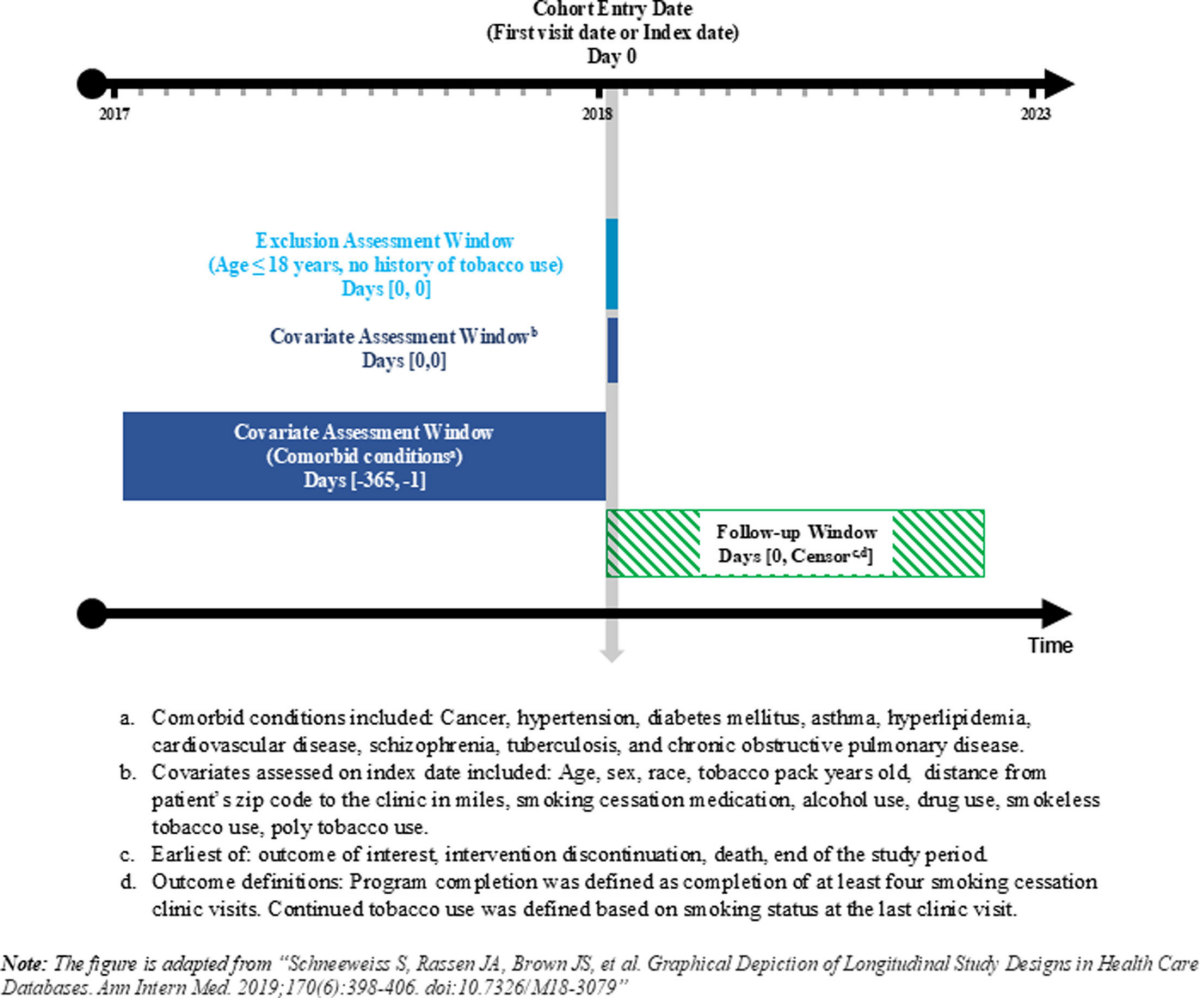
Study design for participants included in the electronic health record-based analysis of the smoking cessation referral program at a medical center in West Virginia, 2018–2023 (N=407)

An observational retrospective cohort study design was used, with patients entering the cohort at the initial intervention visit. The baseline characteristics were assessed on the cohort entry date, with comorbidities assessed over a 12 month look-back period. The patients were followed from the cohort entry date until the intervention was discontinued or the end of the study period, whichever came first. Figure 1 shows a diagrammatic representation of the study design.

### Study participants and eligibility criteria

**Figure 2:**
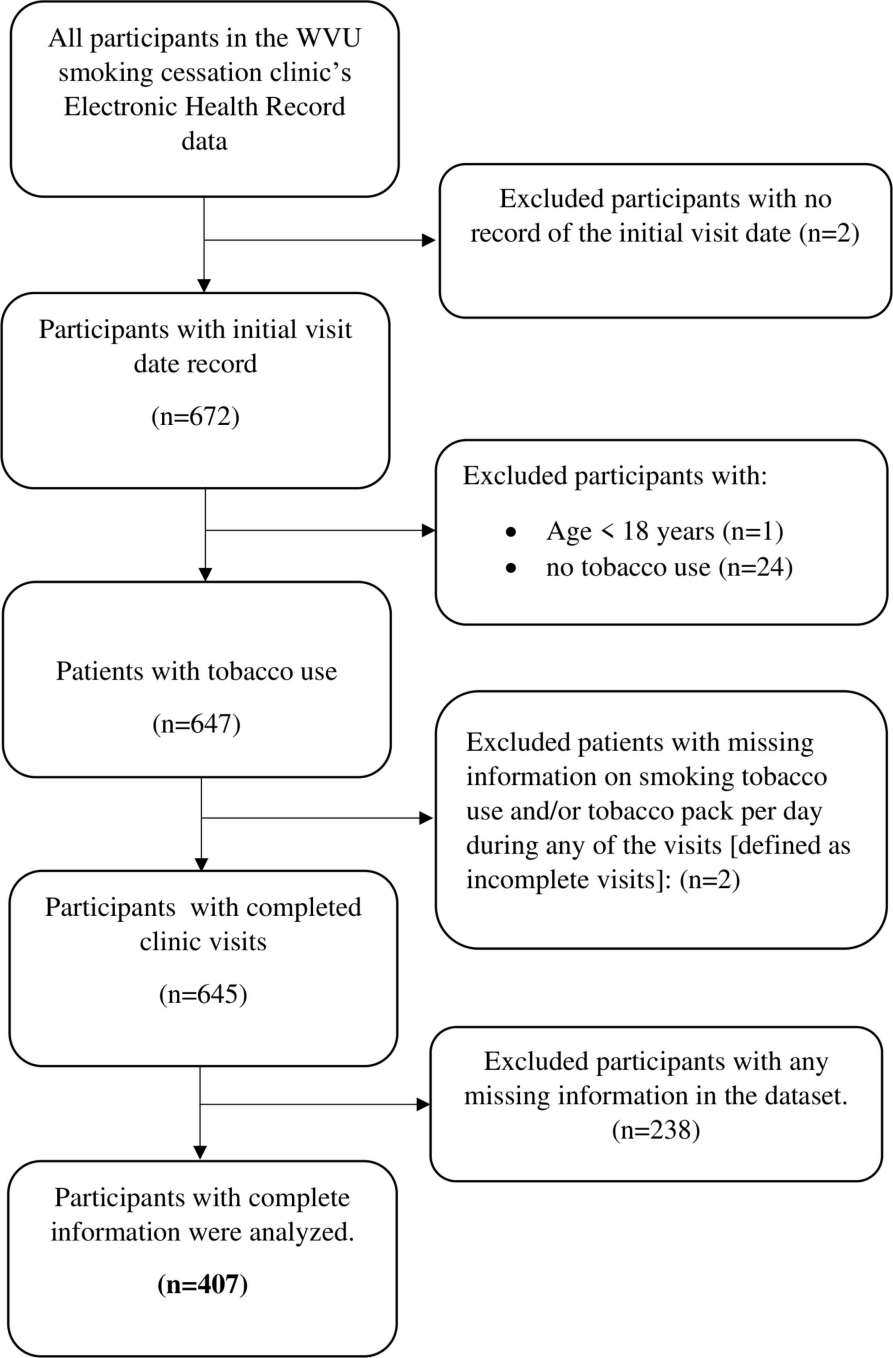
Flow diagram of sample selection for the electronic health record-based analysis of the smoking cessation referral program at a medical center in West Virginia, 2018–2023 (N=407)

Participants aged ≥18 years with a history of tobacco use were included in the study. Participants with missing information on smoking tobacco use and tobacco packs per day during a clinic visit were excluded from the study (37% of the 647 patients were excluded due to missing data). Only complete cases were included in the analysis. Figure 2 shows the sample selection flow diagram.

### Tobacco cessation intervention

Participants entered the tobacco cessation program either through self-referral or provider referral. Intervention was provided via telephonic appointments. Individualized treatment plans consisting of pharmacological and behavioral therapy were given to the patients. Pharmacological treatment was tailored according to the number of cigarettes smoked per day and patient compliance.

During the initial visit, clinicians followed the five As of smoking cessation (Ask, Advise, Assess, Assist, and Arrange) to identify the patient’s willingness to quit and determine the most appropriate individualized plan for them^18^. The clinician began by assessing the patient’s current tobacco status and pack-years history. Next, the patients were educated on the individual health benefits of quitting tobacco use, the risks of continued use, and the harms of exposing others to secondhand smoke. Patients who felt ready to make a quit attempt were offered medication and counseling. A quit date of at least 14 days in the future was encouraged to increase patient’s readiness, reduce withdrawal symptoms, support development of a weaning schedule, and allow time to identify and avoid triggers as they arise. For patients not yet ready to quit, clinicians provided interventions to enhance their readiness for when they are ready to quit.

For patients with previous unsuccessful quit attempts, the clinicians asked, ‘Do you feel you were as ready to quit back then compared to now?’. The clinician then discussed the seven FDA-approved medications, which included five forms of nicotine replacement therapy (NRT) – the patch, gum, inhaler, nasal spray, and lozenge – as well as the two non-NRT medications, bupropion SR, and varenicline. NRTs were available to the patient at any time without the need for a prescription. Patients could also call to connect to the West Virginia tobacco quitline (1–800-QUITNOW) for free counseling and medication assistance, depending on the patient’s insurance coverage.

After the initial visit, three follow-up visits were scheduled at intervals of 4–6 weeks or more frequently as needed. During the follow-up visits, the clinician reassessed the patient’s cessation progress, inquired about any bothersome withdrawal symptoms or cravings, their wellbeing, and medication effectiveness, and, if needed, made changes to the treatment plan. The clinician also discussed lung cancer screening and, if appropriate, ordered a low-dose CT scan of the chest to be performed at the patient’s nearest approved screening facility.

### Independent variables

Age, tobacco pack-years, distance from the patient’s zip code to the clinic^19^, and number of clinic visits were reported as continuous variables. The number of clinic visits was defined as the total number of smoking cessation clinic visits with documented smoking tobacco use and tobacco packs per day information during the study period. The distance from the clinic was calculated by subtracting the difference between the patient’s and the clinic’s zip codes. Baseline comorbidities such as cancer and hypertension were identified using the International Classification of Diseases Clinical Modification (ICD-CM), 10th Revision codes (see Supplementary file Table 1)^12^. The categorical variables were grouped as follows: 1) sex: male and female; 2) race: White and non-White; 3) prescribed smoking cessation medication: nicotine, varenicline, bupropion, and off-label (clonidine or nortriptyline); 4) smokeless tobacco use: never, former, and current smoker; and 5) poly tobacco use: one and two or more, as well as yes and no categories for alcohol use, drug use, and comorbidities.

### Dependent variables

Follow-up for the outcome assessment began the day after the cohort entry date. Program completion was operationalized based on the number of clinic visits. The intervention protocol required patients to complete at least four clinic visits. In addition, the 2015 U.S. Preventive Services Task Force recommendations state that individuals who use tobacco products should receive two quit attempts per year, with each attempt including at least four counseling sessions for cessation per quit attempt. These recommendations were incorporated into the Patient Protection and Affordable Care Act, which requires insurance plans to cover two quit attempts per year, each including up to four counseling sessions^20,21^. Therefore, program completion (yes, no) was defined as completing at least four visits in alignment with the program plan. Continued tobacco use was assessed as a binary variable based on smoking status at the last visit date. Participants who did not continue tobacco use at the last visit were considered successful quitters.

### Statistical analysis

Mean and standard deviation are reported for continuous variables, and frequency with percentage for categorical variables. Differences in categorical variables between the groups were assessed using the chi-squared test, while differences in the continuous variables were compared using a t-test. To determine the predictors of program completion and continued tobacco use on the last visit date, univariable (unadjusted) and multivariable (adjusted) logistic regression analyses were conducted. Odds ratios and p-values were reported for all the regression models. Statistical significance was set at a p<0.10 for the unadjusted models. Due to the low prevalence of program non-completers and successful quitters, only variables meeting this threshold in univariable analyses were included in multivariable models to maintain the recommended ten events-per-variable ratio for adequate power of the study^22-24^. Hence, the adjusted model (AOR) for program completion included age, tobacco pack-years, distance from the patient’s zip code to the clinic, alcohol use, cancer, and hypertension. The adjusted model (AOR) for continued tobacco use at the last visit included tobacco pack-years and the number of clinic visits. Statistical significance in the adjusted models was set at p<0.05. All data management and analyses were conducted using SAS version 9.4 (SAS Institute Inc., Cary, NC).

## Results

Table 1 shows the baseline characteristics of the study participants categorized by the outcome variables. Of the 407 participants in the final dataset, 346 (85%) had at least four clinic visits. However, 333 (81.8%) continued tobacco use on the last visit. In the overall cohort, the mean age of the participants was 55.6 (SD=11.3) years, and the mean tobacco pack-years was 49.8 (SD=29.5). Most participants were females (58%), White (99.3%), and prescribed nicotine for smoking cessation (70.27%). Alcohol use was reported by 29.5% of participants, while 13.3% reported drug use. Additionally, 34.9% had cancer, and 45.2% had hypertension.

**Table 1:** Baseline characteristics of participants included in the electronic health record-based analysis of the smoking cessation referral program at a medical center in west Virginia, 2018–2023 (N=407)

Characteristics	Total n (%)	Program completion^a^			Continued tobacco use on the last visit^b^		
		Yes n (%)	No n (%)	p	Yes n (%)	No n (%)	p
**Total**, n	407	346	61		333	74	
**Age** (years), mean (SD)	55.6 (11.3)	56.3 (11)	51.9 (12.2)	<0.01	55.2 (11.5)	57.3 (10.2)	0.2
**Tobacco pack-years**, mean (SD)	49.8 (29.5)	51 (28.8)	43.5 (33.1)	0.07	51.2 (30.9)	43.9 (21.8)	0.05
**Distance from patient’s zip code to the clinic** (miles), median (IQR)	35.5 (40.9)	19.20 (34.9)	19.9 (49.7)	0.07	19 (35.5)	32.3 (46.1)	0.27
**Number of clinic visits**, mean (SD)	13.6 (10.2)	15.56 (9.8)	2.7 (1)	<0.01	13.1 (10.3)	15.8 (13.7)	0.04
**Biological sex**				0.46			0.59
Male	171 (42)	148 (42.8)	23 (37.7)		142 (42.8)	29 (39.2)	
Female	236 (58)	198 (57.2)	38 (62.3)		191 (57.4)	45 (60.8)	
**Race**				0.47			0.03
White	404 (99.3)	343 (99.1)	61 (100)		332 (99.7)	72 (97.3)	
Non-White	3 (0.7)	3 (0.9)	0 (0)		1 (0.3)	2 (2.7)	
**Smoking cessation medication**				0.47			0.22
Bupropion	37 (9.1)	31 (9)	6 (9.8)		29 (8.7)	8 (10.8)	
Off label	18 (4.4)	17 (4.9)	1 (1.6)		18 (5.4)	0 (0)	
Nicotine replacement therapy	286 (70.3)	245 (70.8)	41 (67.2)		232 (69.7)	55 (73)	
Varenicline	66 (16.2)	53 (15.3)	13 (21.3)		54 (16.2)	12 (16.2)	
**Alcohol use**				0.02			0.28
Yes	120 (29.5)	94 (27.2)	26 (42.6)		102 (30.6)	18 (24.3)	
No	287 (70.5)	252 (72.8)	35 (57.4)		231 (69.4)	56 (75.7)	
**Drug use**				0.65			
Yes	54 (13.3)	47 (13.6)	7 (11.5)		43 (12.9)	11 (14.9)	0.65
No	353 (86.7)	299 (86.4)	54 (88.5)		290 (87.1)	63 (85.1)	
**Smokeless tobacco use**				0.32			0.51
Never	366 (89.9)	309 (89.3)	57 (93.4)		301 (90.4)	65 (87.8)	
Ever	41 (10.1)	37 (10.7)	4 (6.6)		32 (9.6)	9 (12.2)	
**Poly tobacco use**				0.43			0.21
1	377 (92.6)	319 (92.2)	58 (95.1)		311 (93.4)	66 (89.2)	
≥2	30 (7.4)	27 (7.8)	3 (4.9)		22 (6.6)	8 (10.8)	
**Cancer**				<0.01			0.26
Yes	142 (34.9)	131 (37.9)	11 (18)		112 (33.6)	30 (40.5)	
No	265 (65.1)	215 (62.1)	50 (82)		221 (66.4)	44 (59.5)	
**Hypertension**				<0.01			0.71
Yes	184 (45.2)	171 (49.4)	13 (21.3)		152 (45.7)	32 (43.2)	
No	223 (54.8)	175 (50.6)	48 (78.7)		181 (54.4)	42 (56.8)	

a Program completion was defined as completion of at least four smoking cessation clinic visits.

b Continued tobacco use was defined based on smoking status at the last visit date. Statistical significance was set at p<0.05.

### Program completion

Participants who completed at least four visits were older than those who did not complete the cessation program (56.3 vs 51.9 years, p<0.01). A significantly higher proportion of program completers had cancer (37.9% vs 18%, p<0.01) and hypertension (49.4% vs 21.3%, p<0.01), whereas alcohol use was less among completers, compared with non-completers (27.17% vs 42.62%, p=0.02).

Table 2 presents the unadjusted and adjusted results from the logistic regression analyses. In the unadjusted analyses, age, tobacco pack-years, distance from the patient’s zip code to the clinic, alcohol use, cancer, and hypertension were associated with program completion at a statistical significance threshold of p<0.10. In the adjusted analyses, participants with alcohol use had lower odds of program completion compared to those without alcohol use (AOR=0.52; 95% CI: 0.29–0.93; p=0.03). Participants with cancer had higher odds of program completion than those without cancer (AOR=2.77; 95% CI: 1.36–5.65; p=0.01). Similarly, participants with hypertension had higher odds of program completion than those without hypertension (AOR=3.26; 95% CI: 1.64–6.47; p=0.001).

**Table 2:** Adjusted and unadjusted logistic regression analyses of predictors of program completion and continued tobacco use at the last visit among study participants included in a smoking cessation referral program at a medical center in West Virginia, 2018–2023 (N=407)

Characteristics	Program completion^a^				Continued tobacco use on the last visit^b^			
	Unadjusted^c^		Adjusted^d^		Unadjusted^c^		Adjusted^d^	
	OR (95% CI)	p	AOR (95% CI)	p	OR (95% CI)	p	AOR (95% CI)	p
Program completion (ref. no)					0.54 (0.24–1.24)	0.15		
Age (years)	1.03 (1.01–1.06)	0.01	1.01 (0.98–1.04)	0.47	0.98 (0.96–1.01)	0.15		
Tobacco pack-years	1.01 (0.99–1.02)	0.07	1.0 (0.99–1.02)	0.56	1.01 (1.00–1.02)	0.06	1.01 (1.0–1.02)	0.049
Distance from patient’s zip code to the clinic	0.995 (0.99–1.0)	0.08	0.996 (0.990–1.0)	0.20	0.997 (0.991–1.0)	0.27		
Number of clinic visits^e^					0.98 (0.95–0.99)	0.04	0.98 (0.95–0.99)	0.04
Female (ref. male)	0.81 (0.46–1.42)	0.46			0.87 (0.52–1.45)	0.59		
**Smoking cessation medication** (ref. varenicline)								
Bupropion	1.27 (0.44–3.67)	0.52			0.81 (0.30–2.2)	0.96		
Off label	4.17 (0.51–34.2)	0.24			>999.99 (<0.001 – >999.99)	0.96		
Nicotine replacement therapy	1.47 (0.74–2.93)	0.68			0.96 (0.48–1.91)	0.96		
**Substance use**								
Poly tobacco use ≥2 (ref. 1)	1.64 (0.48–5.57)	0.43			0.58 (0.25–1.37)	0.21		
Alcohol use (ref. no)	0.50 (0.29–0.88)	0.02	0.52 (0.29–0.93)	0.03	1.37 (0.78–2.45)	0.28		
Drug use (ref. no)	1.21 (0.52–2.82)	0.65			0.85 (0.42–1.74)	0.65		
**Comorbidities**								
Cancer (ref. no)	2.77 (1.39–5.51)	0.004	2.77 (1.36–5.65)	0.01	0.74 (0.44–1.25)	0.26		
Hypertension (ref. no)	3.61 (1.89–6.90)	<0.001	3.26 (1.64–6.47)	0.001	1.10 (0.66–1.83)	0.71		

a Program completion was defined as completion of at least four smoking cessation clinic visits.

b Continued tobacco use was defined based on smoking status at the last visit date.

c Statistical significance was set at p<0.10 in the univariable (unadjusted) models.

d Only variables with p<0.10 in univariable (unadjusted) logistic regression were included in multivariable (adjusted) logistic regression with statistical significance set at p<0.05.

e Number of clinic visits was excluded from the logistic regression analyses for program completion because it was used to define the outcome and could result in unstable estimates.

AOR: adjusted odds ratio.

### Continued tobacco use at last visit

Participants who continued tobacco use at the last visit had higher tobacco pack-years compared to those who did not continue tobacco use (51.2 vs 43.9 pack-years, p=0.05). In addition, participants who continued tobacco use attended fewer clinic visits compared to successful quitters (13.1 vs 15.8 visits, p=0.04). A lower proportion of non-White participants continued tobacco use at the last visit compared with successful quitters (0.3% vs 2.7%, p=0.03). However, this finding should be interpreted with caution because of the smaller number of non-White participants (n=3) in the study population.

In the unadjusted logistic regression analyses (Table 2), tobacco pack-years and number of clinic visits were associated with continued tobacco use at a statistical significance threshold of p<0.10. Although not statistically significant, the point estimate suggests lower odds of continued tobacco use at the last visit among smoking cessation program completers compared to non-completers (OR=0.54; 95% CI: 0.24–1.24, p=0.15). In the adjusted model, higher tobacco pack-years were associated with higher odds of continued tobacco use at the last visit (AOR=1.01; 95% CI: 1.0–1.02, p=0.049). Whereas a higher number of clinic visits was associated with lower odds of continued tobacco use at the last visit (AOR=0.98; 95% CI: 0.96–0.99, p=0.04).

## Discussion

The current study aimed to determine the predictors of program completion and continued tobacco use at the last visit in a smoking cessation clinic at an academic medical center in West Virginia. Our study showed that, out of the 407 participants in the final dataset, 85% completed at least four clinic visits (program completers). However, 82% still continued using tobacco at their last clinic visit. In the adjusted logistic regression models, alcohol use, cancer, and hypertension were statistically significant predictors of program completion, whereas tobacco pack-years and number of clinic visits were significant predictors of continued tobacco use at the last visit. Despite the high rate of program completion, most patients were still using tobacco on their last visit. This discrepancy suggests that completing the recommended clinic visits may not necessarily translate into cessation success. In our analysis, program completion was associated with lower odds of continued tobacco use, but this association was not statistically significant. Several factors may contribute to the high patient retention but low cessation success in the program, such as the severity of nicotine addiction or dependence, psychosocial factors such as stress and anxiety, and co-occurring substance use.

The literature on predictors of smoking cessation program completion is limited, making direct comparisons with the previous studies challenging. Alcohol consumption in tobacco cessation programs has frequently been studied as a predictor of quit attempts and not program completion. Similar to our findings, a systematic review showed that alcohol consumption negatively impacts the success of a smoking cessation attempt^25^. The systematic review also reported that heavy drinkers have a high smoking relapse risk. A cross-sectional study conducted in England showed that smokers with high-risk alcohol consumption who attempted to quit tobacco were also more likely to try to reduce alcohol use than those who did not attempt to quit tobacco^26^, suggesting a potential increase in successful quit rates with multifactorial interventions that target multiple tobacco and substance use behaviors.

Chronic conditions such as cancer and hypertension were identified as predictors of program completion, a finding observed in previous studies with literature mostly focused on quit outcomes. For example, a study conducted in South Carolina, showed that in an inpatient tobacco treatment program, patients with cancer placed significantly higher levels of importance on quitting smoking compared to those without cancer^27^. Another study showed that patients with chronic conditions like hypertension reported a greater desire and need for assistance with smoking cessation compared to their matched healthy controls^13^. The literature also suggests that comorbidities like cancer and hypertension create an opportunity referred to as a ‘teachable moment’, during which time patients are motivated to undertake risk-reducing health behaviors^28-30^. Hence, cancer and hypertension diagnoses can be used as valuable opportunities for promoting smoking cessation behaviors.

Furthermore, the study identified tobacco pack-years and the number of clinic visits as predictors of continued tobacco use at the last visit in the adjusted analyses. These findings are consistent with previous literature, which has shown that higher smoking intensity and duration are associated with lower smoking cessation success, whereas patients who attended more follow-up visits have higher rates of quit outcomes. For example, a study by Yang et al.^31^ conducted in Korea reported lower odds of successful smoking cessation with increasing smoking duration, smoking dose per day, and lifetime tobacco exposure^31^. Similarly, a study conducted in Denmark reported decreased tobacco abstinence with increasing smoking severity in a dose-dependent manner among smokers attending an intensive smoking cessation program^32^. In addition, a study conducted by Yu et al.^33^ reported that participants with a greater number of visits to the smoking cessation clinic had a lower risk of relapse (HR=0.274; p=0.0124).

### Strengths and limitations

To our knowledge, this is one of the few studies to examine the predictors of smoking cessation program completion in a real-world clinical setting. Second, the longitudinal study design allows assessment of smoking status over multiple clinic visits. Third, we utilized data from electronic health records, which allowed us to record clinical information such as chronic conditions. Fourth, the study was conducted in West Virginia, which is one of the states with the highest tobacco prevalence rates in the US.

Despite its strengths, our study is not without limitations. First, this was a single-center study, which may limit the generalizability of results to other settings or populations. Second, smoking status was self-reported and may be subject to recall bias and social desirability bias. Third, selection bias may be present because the study utilized a clinic-based sample. Fourth, participants with missing data were excluded, and only complete cases were included in the analyses; therefore, if the missing data were not completely at random, selection bias may have been introduced. Fifth, due to the observational study design, temporal and causal relationships between predictors and outcomes cannot be established. Sixth, program completion was defined as attendance at least four clinic visits, which may reflect adherence to the intervention rather than a clinical outcome. Seventh, we were unable to assess whether participants used the smoking cessation medication as prescribed, which could have impacted the study outcomes. Lastly, residual confounding may be present because factors such as nicotine dependence, readiness to quit, program quality, stress, and socioeconomic variables such as marital status, social support, employment, household environment, and education could impact the study results and were not available in the dataset^34^.

### Implications

This study may have important implications for policy, clinicians, and research. Since adults who have a greater number of comorbidities may have a higher likelihood of quitting tobacco, policies should promote the development of cessation programs tailored to this population. Furthermore, the findings also suggest the potential value of integrating tobacco cessation with alcohol reduction strategies to improve quit success. Clinicians should actively screen patients for chronic conditions and leverage these conditions as motivations for smoking cessation. Lastly, the low cessation rate despite high program completion highlights a critical gap and warrants further research. Future studies should explore factors contributing to this discordance, which will be essential for guiding more effective and comprehensive cessation interventions.

## Conclusions

Alcohol use, cancer, and hypertension were identified as predictors of program completion, while tobacco pack-years and the number of clinic visits were predictors of continued tobacco use on the last clinic visit. Although most participants completed at least four visits, cessation rates remained low. This finding underscores the need for further research to understand factors contributing to this discordance, including broader sociodemographic and behavioral variables. Our findings may also suggest integrating tobacco and alcohol cessation strategies and leveraging chronic disease diagnosis as a motivation in interventions to support quit outcomes.
