# Supplementary material for: Determinants of smoking cessation program completion and continued tobacco use in a referral program in West Virginia: An EHR data analysis from 2018–2023
Source: Tob Prev Cessat. 2026 Jul 9;12:10.18332/tpc/221805. doi: 10.18332/tpc/221805 (PMC13352313; doi:10.18332/tpc/221805)
Supplement: Supplementary file Material 1 [file TPC-12-33-s001.pdf]

Supplementary material

Supplementary table 1: ICD-10-CM Codes for identifying comorbidities.

Disease/illness/ disorder	ICD-10-CM codes
Hypertension	I10, I11, I12, I13, I15, I16
Cancer	C00, C01, C02, C03, C04, ,C05, C06, C07,C08, C09, C10, C11, ,C12,C13, C14, C15, C16, C17, C18, C19, C20, C21, C22, C23, C24, ,C25, C26, C30, C31, C32, C33,C34, C37,C38, C39, C40, C41, C43 ,C44 ,C45,C46, C47, C48, C49, C50, C51, C52,C53, C54, C55,C56,C57, C58,C60, C61,C62, C63, C64,C65,C66,C67, C68, C69, C70, C71, C72, C73,C74, C75, C76, C77, C78, C79, C80, C81, C82, C83, C84, C85, C86, C88, C90, C91 ,C92, C93, C94, C95, C96, C97,D00, D01, D02, D03, D04, D05, D06, D07, D09, D10, D11, D12, D13, D14, D15, D16, D17, D18, D19, D20, D21, D22, D23, D24,D25, D26, D27,D28, D29, D30, D31, D32, D33, D34,D35, D36, D37, D38, D39, D40, D41, D42, D43, D44, D45,D46, D47, D48

STROBE Statement—checklist of items that should be included in reports of observational studies

	Item No.	Recommendation	Page No.	Relevant text from manuscript
**Title and abstract**	1	(*a*) Indicate the study's design with a commonly used term in the title or the abstract	Page # 2	"An observational retrospective cohort study analyzed electronic health records (2018-2023) collected from a tobacco cessation clinic in WV…"
		(*b*) Provide in the abstract an informative and balanced summary of what was done and what was found	Page # 2 to 3	"…Logistic regression estimated odds ratio (OR) and 95% confidence intervals (CI) for predictors of program completion and continued tobacco use….." "….Age (OR: 1.03, 95% CI: 1.01-1.06), cancer (2.89, 1.41-5.95), and hypertension (3.53, 1.75-7.11) were associated with higher odds of program completion…."
**Introduction**				
Background/rationale	2	Explain the scientific background and rationale for the investigation being reported	Page # 4 to 5	"…Numerous studies have assessed factors associated with successful tobacco cessation in the U.S. and other countries.[6–10] For example, Lee et al.."

				"…While several studies reported on factors associated with tobacco cessation, few have examined predictors of completion of clinic-based cessation programs in the U.S.[16] Program completion reflects patients' participation and retention within the cessation program. Although literature.."
Objectives	3	State specific objectives, including any prespecified hypotheses	Page # 6	"..Building on this limited but growing evidence, the present study aims to address two questions within a WV-based smoking cessation clinic. Using a retrospective cohort design, the study aims to determine 1) the predictors of completion of the tobacco cessation program, defined as completing more than four visits to the cessation clinic, and 2) the predictors of smoking cessation failures, defined as continued tobacco use at the last visit date to the cessation program…"
**Methods**				
Study design	4	Present key elements of study design early in the paper	Page # 7	"An observational retrospective cohort study design was utilized in which patients entered the cohort at the initial intervention visit. The baseline characteristics

				were assessed on the cohort entry date,…"
Setting	5	Describe the setting, locations, and relevant dates, including periods of recruitment, exposure, follow-up, and data collection	Page # 7 and 8	"The data for this analysis was obtained from the electronic health records of the people referred to the tobacco cessation clinic at the Mary Babb Randolph Cancer Center in Morgantown, WV, between 2018 and 2023.."<br><br>"Participants entered the tobacco cessation program either through self-referral or provider referral. The clinic visits were scheduled in person.."
Participants	6	(*a*) *Cohort study*—Give the eligibility criteria, and the sources and methods of selection of participants. Describe methods of follow-up<br>*Case-control study*—Give the eligibility criteria, and the sources and methods of case ascertainment and control selection. Give the rationale for the choice of cases and controls<br>*Cross-sectional study*—Give the eligibility criteria, and the sources and methods of selection of participants	Page # 7	"Participants aged 18 years and above with a history of tobacco use were included in the study. Participants with missing information on smoking tobacco use and tobacco packs per day during a clinic visit were excluded.."
		(*b*) *Cohort study*—For matched studies, give matching criteria and number of exposed and unexposed<br>*Case-control study*—For matched studies, give matching criteria and the number of controls per case	-	
Variables	7	Clearly define all outcomes, exposures, predictors, potential confounders, and effect modifiers. Give diagnostic criteria, if applicable	Page # 9 and 10	"Age, tobacco pack years old, distance from patient's zip code to the clinic, and number of completed clinic visits

				were reported as continuous variables…”
Data sources/ measurement	8*	For each variable of interest, give sources of data and details of methods of assessment (measurement). Describe comparability of assessment methods if there is more than one group	Page # 9 and 10	“Follow-up for the outcome assessment began the day after the cohort entry date. Program completion was operationalized based on the…”
Bias	9	Describe any efforts to address potential sources of bias	-	
Study size	10	Explain how the study size was arrived at	**Figure 2**	**Figure 2** shows the study flow diagram

Continued on next page

Quantitative variables	11	Explain how quantitative variables were handled in the analyses. If applicable, describe which groupings were chosen and why	Page # 9 and 10	"The categorical variables were grouped as follows: (i) sex: male and female..."
Statistical methods	12	(*a*) Describe all statistical methods, including those used to control for confounding	Page # 10	"Mean and standard deviation were reported for continuous variables, and frequency with percentage for categorical variables. Differences in categorical variables between the groups were assessed using the Chi-square test.."
		(*b*) Describe any methods used to examine subgroups and interactions	-	
		(*c*) Explain how missing data were addressed	-	
		(*d*) *Cohort study*—If applicable, explain how loss to follow-up was addressed<br>*Case-control study*—If applicable, explain how matching of cases and controls was addressed<br>*Cross-sectional study*—If applicable, describe analytical methods taking account of sampling strategy	-	
		(*e*) Describe any sensitivity analyses	-	
**Results**				
Participants	13*	(a) Report numbers of individuals at each stage of study—eg numbers potentially eligible, examined for eligibility, confirmed eligible, included in the study, completing follow-up, and analysed	Figure 2	Figure 2
		(b) Give reasons for non-participation at each stage	-	
		(c) Consider use of a flow diagram	Figure 2	Figure 2
Descriptive data	14*	(a) Give characteristics of study participants (eg demographic, clinical, social) and information on exposures and potential confounders	Page # 11	"**Table 1** shows the characteristics of the patients in the groups categorized by the outcome variables. Of the 407 participants in the final dataset, 346 (85.01%) had >4 clinic visits. However, 333 (81.82%).."
		(b) Indicate number of participants with missing data for each variable of interest	-	
		(c) *Cohort study*—Summarise follow-up time (eg, average and total amount)	-	
Outcome data	15*	*Cohort study*—Report numbers of outcome events or summary measures over time	-	
		Case-control study—Report numbers in each exposure category, or summary measures of exposure	-	
		Cross-sectional study—Report numbers of outcome events or summary measures	-	

Main results	16	(*a*) Give unadjusted estimates and, if applicable, confounder-adjusted estimates and their precision (eg, 95% confidence interval). Make clear which confounders were adjusted for and why they were included	Page # 11 and 12	"**Table 2** presents the unadjusted and adjusted results for the regression model. The model was adjusted for age…"
		(*b*) Report category boundaries when continuous variables were categorized	-	
		(*c*) If relevant, consider translating estimates of relative risk into absolute risk for a meaningful time period	-	

Continued on next page

Other analyses	17	Report other analyses done—eg analyses of subgroups and interactions, and sensitivity analyses	-	
**Discussion**				
Key results	18	Summarise key results with reference to study objectives	Page # 13	"The current study aimed to determine the predictors of program completion and continued tobacco use at the last visit in a smoking cessation clinic at an academic medical center in WV. Our study showed that,.."
Limitations	19	Discuss limitations of the study, taking into account sources of potential bias or imprecision. Discuss both direction and magnitude of any potential bias	Page # 15	"Despite its strengths, our study is not without limitations. We were unable to assess whether the participants used the smoking cessation medication as prescribed to them, which could impact the study outcomes…"
Interpretation	20	Give a cautious overall interpretation of results considering objectives, limitations, multiplicity of analyses, results from similar studies, and other relevant evidence	Page # 13 to 16	"In conclusion, age, alcohol use, cancer, and hypertension were identified as predictors of program completion while age and tobacco pack years old were predictors of continued tobacco use on the last clinic visit..."
Generalisability	21	Discuss the generalisability (external validity) of the study results	Page # 15	"First, this was a single center study which may limit the generalizability of results to other settings or populations."
**Other information**				
Funding	22	Give the source of funding and the role of the funders for the present study and, if applicable, for the original study on which the present article is based	Page # 17	There was no financial assistance for the author(s)' research, authorship, or publication of this article.

*Give information separately for cases and controls in case-control studies and, if applicable, for exposed and unexposed groups in cohort and cross-sectional studies.

Note: An Explanation and Elaboration article discusses each checklist item and gives methodological background and published examples of transparent reporting. The STROBE checklist is best used in conjunction with this article (freely available on the Web sites of PLoS Medicine at http://www.plosmedicine.org/, Annals of Internal Medicine at http://www.annals.org/, and Epidemiology at http://www.epidem.com/). Information on the STROBE Initiative is available at www.strobe-statement.org.

© 2026 Pathan M. et al.

SUPPLEMENTARY FILE DISCLAIMER

The content has been provided by the author(s) and has not been reviewed, verified, or endorsed by European Publishing. It may not have undergone peer review. The views, opinions, and recommendations expressed are solely those of the author(s) and do not necessarily reflect the position of European Publishing. European Publishing accepts no responsibility or liability for any consequences arising from the use of, or reliance on, this content.
